# Research on Optimization Model of Multisource Traffic Information Collection Combination Based on Genetic Algorithm

**DOI:** 10.1155/2022/3793996

**Published:** 2022-01-27

**Authors:** Jianwei Guo, Yongbo Lv

**Affiliations:** School of Traffic and Transportation, Beijing Jiaotong University, Beijing 100044, China

## Abstract

In order to reduce the excessive use of multisource traffic information collection system, a multisource traffic information collection combination optimization mode is proposed based on genetic algorithm in this paper. This model is mainly used to analyze the traffic management data in the city. According to the collected data information, the characteristics of the traffic equipment can be effectively analyzed. Basing on the market demand and supply relationship, the multisource traffic information collection combination optimization model is used to complete the reorganization and optimization of the traffic information in this paper, to acquire the main convolution feature variables of the model. The data information combination processing is performed according to the acquired feature variables, and the genetic algorithm is used to adjust the multisource traffic information. During the process of information fusion data analysis, the multisource traffic information clustering and fuzzy constraint control can be performed effectively to realize the optimization of the team's traffic information collection combination. Finally, the simulation results show that the method proposed in this paper is more accurate in realizing the optimization process of multisource traffic information collection and combination and has a better degree of information fusion.

## 1. Introduction

At present, many large-scale cities in China have gradually increased their investment in urban transportation systems, especially in the collection of multisource traffic information [1, 2], wasting a lot of manpower and financial resources. as the foundation of urban traffic infrastructure, multisource traffic information can be used to effectively improve the During accuracy and efficiency of traffic information collection, laying the foundation for improving the urban intelligent traffic system [3, 4]. However, currently, due to the single structure of the existing acquisition system hardware in the use process, the collected data information is inaccurate, and the cost of investment is huge. The limitation of the single-structured traffic data acquisition equipment lies in the small acquisition range. Fixed information collection period and fixed types of acquired data information, resulting in large-scale investment and serious waste of resources. Meanwhile, all kinds of information collection equipment are damaged seriously leading to huge maintenance costs. The use of different types of information collection equipment will cause repeated collection of traffic information and increase the workload.

Based on genetic algorithm, a combined model of multisource traffic information collection is proposed in this paper. The model can be used to effectively analyze the results of the obtained multisource traffic information, genetic algorithms are combined to collect and combine the initial traffic information, complete the reconstruction of the multisource traffic information spatial structure, and obtain the convolutional characteristics of the traffic information. According to the characteristic variables, the fusion processing and combination of data information can be carried out. Complete distributed clustering of multisource information in the fusion space. Finally, the analysis of experimental simulation results shows that the method in this paper has superior performance and information collection ability in the process of extracting multisource traffic information collection combination.

## 2. Information Collection and Feature Preprocessing

### 2.1. Collection Combination Optimization System

**Figure 1:**
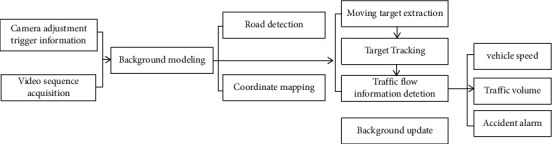
System flow chart.

The flow of mobile positioning and real-time monitoring system of road vehicle is shown in Figure 1, which mainly includes three parts: extraction and tracking of vehicle targets, road condition detection and real-time monitoring of traffic flow [5, 6]. The extraction and location of vehicle targets are mainly realized by the construction of the detection background to extract the characteristics of the moving vehicle, while monitoring the vehicle parameters in the implementation. The road background is based on for modeling the road condition monitoring, and the road surface can be divided by the acquired data information, and the vehicle detection area is extracted to obtain the conversion between the road plane coordinates and the left side of the multimedia spectrum plane. The data of the road and traffic flow obtained in previous times is integrated by the road traffic to complete the data analysis and statistics of the traffic flow on different lanes [7–11]. This system uses the traffic flow pictures collected by the cameras placed on the platform.

The main control software design is mainly composed of two main programs, which are the main control programs that can complete different business logics and the programs that can realize the user-side GUI process control. The main control program is mainly to receive external commands, and meanwhile send the obtained traffic flow information to the corresponding business module for processing, effectively realizing the business logic between systems.

The main process uses a message transfer mode including a communication module, a message transfer module, and a business module. The communication module mainly establishes a network link [12–14], and sends the received data information from the control information obtained to an arbitration level information commands are sent to the control center through the service interface. The control command center module executes in accordance with the message queue mechanism acquired by the GUI process system and the method of sending and receiving information between the corresponding processes. When the service module mainly processes specific services and all service modules are initialized, the service function module sends the command information that needs to be processed to the message control module. At the same time, when data information is received, the data control center will send the command information to the business module for sorting. The GUI process is developed based on the embedded graphics library mimugui, which is mainly a graphical user interface that realizes the needs of business logic. The GUI process communicates with the main process in the same way as the message queue [15].

**Figure 2:**
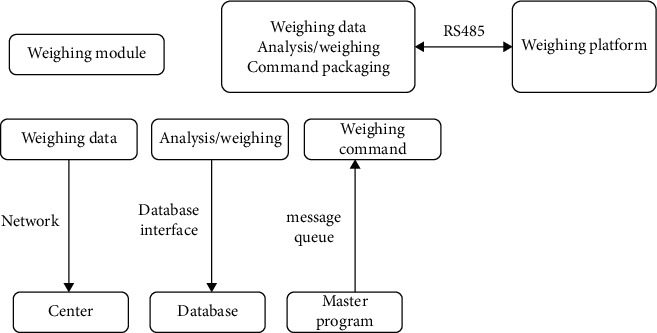
Block diagram of quantity statistics module.

The vehicle positioning detection module mainly communicates with the weight measurement platform and adopts the modbus protocol. This module is the master, and the vehicle volume measurement platform is mainly used as a slave device. The input of the vehicle positioning detection module is received using the RS485 interface, and the vehicle volume detection module is used to extract vehicle volume-related data information, and at the same time, it is sent to the message arbitration module to obtain the quantity statistics operation command from the outside. The output of the detection module includes the transmission of the detection result data to the center via the network. The results of the quantity statistics are stored in the database. The detection of instructions are sent to the detection platform through the RS485 interface. the RS485 interface and the quantity statistics platform are used for the processing of the quantity statistics module to construct information and data associations. Meanwhile, the automatic fixed time is set at the RS485 interface to automatically and regularly read the vehicle information from the quantity statistics platform, and the analysis results are sent to the module for processing through data analysis. The interface to network is used by vehicle positioning detection module to send statistical results to the control center and stores them in the database. The processing steps of the detection module are shown in Figure 2.

**Figure 3:**
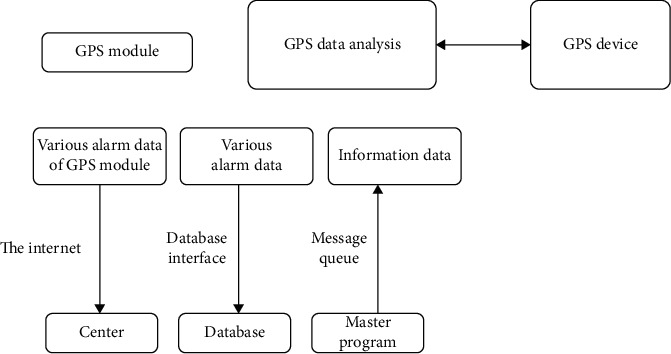
GPS module block diagram.

The GPS module is used to obtain the geographic information parameters of the vehicle in this paper. When the vehicle image is acquired, and it is used in the system *l*, the processing of the above data can be displayed digitally, and the obtained results will be stored. The input GPS device of the GPS module is GPS information data sent via the network in this paper. The output of the GPS module's data information is encrypted and protected in the network center, and then the message arbitration module is used to store the vehicle-related data information in the GUI module. The warning information about GPS is stored in the database, and the data sent is mainly the warning information of GPS. The paper. GPS module is usually processed with a dedicated network to obtain the GPS device data information and use the GPS data analysis function module to realize the transmission of relevant information data and relevant alarm information by the network center GPS. As shown in Figure 3.

The data transmission task mainly includes the vehicle-mounted terminal transmitting data to the monitoring platform. In addition to the real-time data of the vehicle, it also includes sending the identification information and data mentioned in the link management task to the mainly includes branch of monitoring platform as supplement. The vehicle-mounted terminal can transmit information to multiple monitoring centers, so multiple data transmission tasks coexist. The data management is performed for coordinating multiple data transmission tasks to prevent conflicts and resource competition.

### 2.2. Collection of Multisource Traffic Information

**Figure 4:**
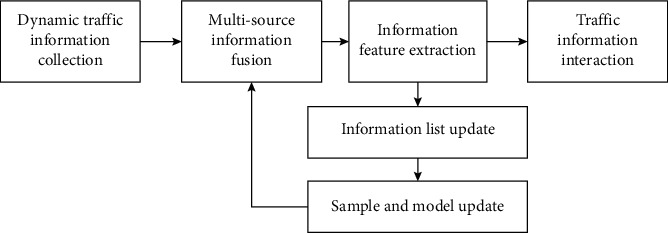
The overall structure model combined by multisource traffic information collection and fusion collection.

In order to realize the optimal collection and fusion dispatching of traffic in a complex traffic network environment, first the design of a distributed data structure of multisource and multisource traffic information shall be carried out in the complex traffic network environment, and a multisource traffic information database structure is built in a complex network environment,..0 + the fuzzy decentralized preservation center of multisource traffic information needs to be represented by 4 groups G. Suppose *i* is the feature space of the interaction of multiple sources of traffic information in a complex traffic network environment. The multisource traffic information is reconstructed in a high-dimensional feature space in a complex traffic network environment using the coupled statistical method of multiple non-linear components, combined with pattern recognition and adaptive clustering methods, to carry out the information fusion and feature extraction of source traffic information in multiple traffic network environments. Based on the above analysis, Figure 4 shows the overall structure model of building a combination of multisource traffic information collection and fusion collection in a complex traffic network environment.

According to Figure 4, the multisource traffic information collection process contains a large amount of interference information and road condition information. This multisource traffic information indicates the data by spectral characteristics. The multisource and multisource traffic information contains *n* variables. {*y*_1_, *y*_2_,…, *y*_*n*_} can be used to represent the variable threshold corresponding to *a*_*j*_. The simplified mathematical model of multisource traffic information can be explained by the following formula.

(1) $$\begin{array}{c} \left\{\begin{array}{c} G_{1}=b_{11}a_{1}+b_{12}a_{2}+,\ldots ,b_{1n}a_{n}\\ G_{2}=b_{21}a_{1}+b_{22}a_{2}+,\ldots ,b_{2n}a_{n}\\ \cdots \\ G_{n}=b_{n1}a_{1}+b_{n2}a_{2}+,\ldots ,+b_{nn}a_{n} \end{array}\right.. \end{array}$$

Among them, *a*_*j*_ and *G*_*k*_ both have a strong correlation, which represents a distributed array of sensors that collect multisource traffic information in a complex traffic network environment, and use multisensor fusion recognition methods for effective information mining in multisource traffic in the Internet of Vehicles. The fuzzy adaptive clustering method is used to construct multisource in multi traffic information statistical feature quantity, combined with normalized feature extraction method, the multisource traffic information autoregressive analysis result is *u*(*x*_*j*_)*ξ*_*j*_, and the detection problem of multisource traffic information is transformed into a linear problem of measuring the dynamic distribution of multisources in a complex traffic network environment. According to the above design, the statistical result model of multisource traffic information is analyzed, and the statistical feature sampling method is used to carry out the acquisition of the information of the original multisource traffic [8].

### 2.3. Big Data Fusion of Traffic Information

Based on the time-frequency coupling distribution of the multisource traffic bit sequence, the genetic algorithm method is used to remove the interference component and the feature set of the multisource traffic information [9] and realize the fuzzy clustering processing of the multisource traffic information of the automobile network: *k*(*x*_*i*_, *x*_*j*_)

(2) $$\begin{array}{c} \left.\begin{array}{c} \underset{w,b,\xi }{\min}\frac{1}{2}{\left\|w\right\|}^{2}+C\sum_{j=1}^{l}u\left(x_{j}\right)\xi_{j}\\ \text{s.t. }y_{j}\left(\left(w\cdot x_{j}\right)+b\right)+\xi_{j}\geq 1\\ \xi_{j}\geq 0,\;j=1,2,\ldots ,l \end{array}\right\}. \end{array}$$

Regarding the nonlinear collection and combination problem, based on the temporal variability and random change characteristics of traffic information, the fuzzy dynamic change kernel function *k*(*x*_*i*_, *x*_*j*_) is introduced for dynamic feature separation. The following quadratic planning shows the information fusion of multisource and multisource traffic information problem.

(3) $$\begin{array}{c} \left.\begin{array}{c} \underset{\alpha }{\min}\frac{1}{2}\sum_{i=1}^{l}\sum_{j=1}^{l}y_{i}y_{j}\alpha_{i}\alpha_{j}K\left(x_{i},x_{j}\right)-\sum_{j=1}^{l}\alpha_{j}\\ \text{s.t. }\sum_{j=1}^{l}y_{j}\alpha_{j}=0\\ 0\leq \alpha_{j}\leq u\left(x_{j}\right)C,\;j=1,2,\ldots ,l \end{array}\right\}. \end{array}$$

Based on the results of feature extraction, statistical regression analysis is used to analyze the nonlinear structure group of multisource traffic information in a complex traffic network environment [10], and a linear coupling model is derived:

(4) $$\begin{array}{c} x_{k}=\sum_{n=0}^{N/2-1}2\left(a_{n}\cos\frac{2\pi kn}{N}-b_{n}\sin\frac{2\pi kn}{N}\right),\;k=0,1,\ldots ,N-1. \end{array}$$

Among them, *a*_*n*_ represents the feature distribution of multisource traffic information linear programming in a complex transportation network environment. There are *m* multisource traffic information nodes *A*_1_, *A*_2_,…, *A*_*m*_ in a complex transportation network environment to construct multisource traffic information fusion scheduling in a complex transportation network environment. Its mathematical expression is as follows:

(5) $$\begin{array}{c} \min\left(f\right)=\sum_{i=1}^{m}\sum_{j=1}^{n}C_{ij}X_{ij},\\ \text{s.t. }\left\{\begin{array}{c} \sum_{j=1}^{m}X_{ij}=a_{i},\;i=1,2,\ldots ,m\\ \sum_{i=1}^{m}X_{ij}=b_{i},\;j=1,2,\ldots ,n\\ X_{ij}\geq 0,\;i=1,2,\ldots ,m;\;j=1,2,\ldots ,n \end{array}\right.. \end{array}$$

Assuming that the number of multisource and multisource traffic information distribution nodes in the current complex traffic network environment is *n*, *N*_1_,…, *N*_*n*_ the learning method of fuzzy convolutional neural network is adopted to realize the big data fusion of traffic information.

## 3. Collection Combination Configuration Model

When analyzing traffic conditions, a sufficient amount of multisource traffic information must be used as a database, but excessive traffic data will cause data duplication and redundancy [5]. Meanwhile, too many information collection devices have limited improvement in real-time monitoring of traffic conditions, which also causes a waste of social resources. Therefore, the basic idea of modeling in this paper is to comprehensively utilize the advantages of multiple multisource traffic information collection devices, make up for the shortcomings of different types of equipment, and form a multi-source traffic information collection system that constitutes an efficient and low-cost combination structure for correct collection and analysis of the data of the communication status.

A single multisource traffic information collection device is the basis of a composite configuration system. In order to make full use of the applicability of a single device, the principle of single configuration is first discussed.

### 3.1. Layout Based on a Single Multisource Traffic Information Collection Device

General information collection devices are considered mainly in this article, which divides the types of devices into fixed and mobile types, and discusses their configuration methods.

#### 3.1.1. Layout Method of Fixed Collection Equipment

Set the road network as G (T, A), where *T* is the node in the road network and A is the road section in the road network. *α*_*ji*_ is defined as whether *i* multisource traffic information collection equipment is installed on the *j* section. If it is set, then *α*_*ji*_=1, if not set, then *α*_*ji*_=0. Based on the maximization of utility, the utility maximization model of collection equipment is established [6]:

(6) $$\begin{array}{c} \max U=\sum_{\alpha_{i}\in A}\alpha_{ji}f_{i}r_{j},\\ \alpha_{ji}=0\text{ or }1, \end{array}$$

where *U* is the utility of the collection equipment; *f*_*j*_ is the traffic fluctuation coefficient of road section *j;* and *r*_*j*_ is the objectively important coefficient of road section *j*.

*(1) Determination of Traffic Fluctuation Coefficientf*_*j*_. The traffic variation coefficients in different sections are different. The traffic variation coefficient is mainly affected by the traffic flow [7].

The traffic fluctuation coefficient is determined by the traffic volume. Take the observation sample *h*_*k*_, *k*=1,2,…, *l*, calculate the sample mean *μ*(*h*_*k*_) variance *μ*(*h*_*k*_), let

(7) $$\begin{array}{c} f_{k}=\frac{\delta \left(h_{k}\right)}{\mu \left(h_{k}\right)}. \end{array}$$

The maximum is taken through computation, then

(8) $$\begin{array}{c} f_{\max}=\max\left(h_{k}\right),\;k=1,2,3,\ldots ,m,\\ f_{\min}=\min\left(h_{k}\right),\;k=1,2,3,\ldots ,m. \end{array}$$

Based on equation (3) (9)

(9) $$\begin{array}{c} \sum_{k=1}^{l}\mu^{2}\left(h_{k}\right)=\sum_{k=1}^{l}\delta^{2}\left(h_{k}\right)/f_{k}^{2}. \end{array}$$

Then,

(10) $$\begin{array}{c} \sum_{k=1}^{l}\delta^{2}\left(h_{k}\right)/f_{\min}^{2}\geq \sum_{k=1}^{l}\mu^{2}\left(h_{k}\right)\geq \sum_{k=1}^{l}\delta^{2}\left(h_{k}\right)/f_{\max}^{2}. \end{array}$$

Due to change in equal change, the following is obtained

(11) $$\begin{array}{c} f_{\max}\geq {\left[\sum_{k=1}^{l}\delta^{2}\left(h_{k}\right)/\sum_{k=1}^{l}\mu^{2}\left(h_{k}\right)\right]}^{1/2}\geq f_{\min}. \end{array}$$

Then the fluctuation coefficient of the road section is

(12) $$\begin{array}{c} f_{j}={\left[\sum_{k=1}^{l}\delta^{2}\left(h_{k}\right)/\sum_{k=1}^{l}\mu^{2}\left(h_{k}\right)\right]}^{1/2}. \end{array}$$

*(2) Determination of the Objective Importancer*_*j*_ *of Road Sections*. The importance of different roads is almost inconsistent. The location of the bottleneck in the road network has a great impact on the road network. For example, sections with heavy traffic during peak hours and sections in busy streets are compared with the section with small traffic volume has relatively little impact on the road network. For example, unimportant sections near the suburbs.

The importance of the road network can be calculated according to the traffic volume [8]. The total traffic flow *Q*=∑_*j*=1_^*r*^*q*_*j*_ is defined, where *r* is the total number of intervals, *q*_*j*_ is the flow of interval *j*, *Q* ≤ *LL* is the network capacity, and *Q* is the historical data. Therefore, the objective importance *T*_*j*_ of segment *j* is

(13) $$\begin{array}{c} r_{j}=\frac{q_{j}}{Q}. \end{array}$$

#### 3.1.2. How to Determine the Sample Size of GPS Floating Car

Different from fixed information collection equipment, mobile equipment mainly adjusts the input amount to ensure sufficient data collection [9]. This article chooses the floating lane network coverage. *γ* as an index to evaluate the adequacy of the input amount, there are

(14) $$\begin{array}{c} \gamma =\left(\frac{r}{R}\right)\times 100\%. \end{array}$$

In the formula: *r* is the number of road sections that can be covered by the floating car and *R* is the total road section number in the road network.

The condition for judging whether a road is covered is that GPS floating car information can be obtained during the research cycle. With reference to previous research experience, there is the following relationship between the number of GPS floating vehicles in the road network and the capture rate.

(15) $$\begin{array}{c} \gamma =\exp\left\{-{\left(a_{0}+a_{1}x_{2}+a_{2}x_{2}+a_{3}x_{3}+a_{4}x_{4}+a_{5}x_{5}+a_{6}x_{6}+a_{7}\ln\left(x_{7}\right)\right)}^{2}\right\},\\ \left\{\begin{array}{c} \gamma \geq C_{1}\\ x_{7}\leq C_{2}\\ \frac{d_{r}}{d_{x_{7}}}\geq C_{3} \end{array}\right., \end{array}$$

where *γ* is the coverage of the road network; *x*1 is the area of road network, 100 km^2^; *x*2 is the ratio of the expressway in the road network to the total road length; *x*3 is the ratio of the main arterial road to the total road length; *x*4 is the secondary arterial road to the total road length. *X*5 is the proportion of the length of secondary access road to the total road; *x*6 is the density of road network, km/km^2^; *x*7 is the number of floating vehicles, (in hundreds); C1 is the minimum threshold of road network coverage, (in hundreds); C2 is maximum number threshold of GPS floating vehicles, (in hundreds); C3 is the minimum threshold of the marginal contribution of the number of floating cars to the coverage of the road network. In the model, a0, a1, a2, a3, a4, a5, a6, and a7 can be obtained by regression analysis.

### 3.2. Combination Configuration Principle for Multisource Traffic Information Collection

The combination configuration of multisource traffic information collection shall satisfy the following principles:

1. Ability to collect more effective multi-source traffic information;
2. While minimizing the cost of the arrangement of the collection equipment, more effective multisource traffic information can be collected, and multifunction can be used to limit the overlap of data.
3. The reduction of equipment input discussed in this paper is mainly based on the viewpoint of reducing the redundancy of data collection and subject to the optimization goals of other functions of the equipment.

### 3.3. Establishment of Model

The set N is defined as the number of different types of multisource traffic information collection devices, and different collection devices can be put on the same road.

The defined variable *a*_*ji*_ indicates whether the *i*th multisource traffic information collection device is installed on the *j*th road. If is set, then *a*_*ji*_=1; if it is not set, then *a*_*ji*_=0, *a*_*ji*_ form matrix A.

#### 3.3.1. Construction of Objective Function considering Economic Benefits

Targeting at the lowest total configuration cost of collection equipment, with collecting the best multisource traffic information as the premise, there are various configuration methods for collection equipment. The cost of different equipment is different. In order to minimize the total cost of configuration, the most suitable layout plan can be obtained by optimizing the combination configuration.

In this article, we only considered general fixed and mobile collection equipment. In the construction of the function for the purpose of economic effect, the key variable of the device needs to be considered for the fixed information collection device, and only the number of equipment needs to be considered for the mobile type.

The definition *φ*_*ji*_ represents the factor variable of *i* kinds of multisource traffic information collection equipment on the *j* section, and the decisive factor is the characteristics of the collection equipment and the characteristics of the road section.

Assuming that there are *r* sections in the road network, the numbers of fixed and mobile equipment are *l* and *e*, respectively. In order to minimize the overall cost of the equipment, the objective function is

(16) $$\begin{array}{c} \min=\sum_{i=1}^{l}\sum_{j=1}^{r}a_{ji}\times \varphi_{ji}\times c_{i}+\sum_{i=1}^{e}n_{i}c_{i}. \end{array}$$

In the formula: the value of *a*_*ji*_ is taken as 1; *c*_*i*_ is the cost of the *i*th type of equipment; and *n*_*i*_ is the number of the *i*th type of equipment.

#### 3.3.2. Construction of Objective Function considering Comprehensive Benefits

In order to meet the basic requirements for collecting data, a multifunctional comprehensive income function is also established to reduce the overall cost of the equipment.

The variables *x*_*ik*_ is defined as (*x*_*ik*_ 0–1 variables), *x*_*ik*_ indicating whether the *k*th data can be obtained by the *i*th multisource traffic information collection device, if it can be obtained, then *x*_*ik*_=1∇(*f*, *f*′), if it cannot be obtained, then *x*_*ik*_=0.

From the above analysis, the following matrix can be obtained:

(17) $$\begin{array}{c} P_{ijk}=\left[A\right]\left(\begin{array}{cccc} x_{11} & x_{12} & \cdots & x_{1g}\\ \vdots & \vdots & \; & \vdots \\ x_{\left(e+l\right)1} & x_{\left(e+l\right)2} & \cdots & x_{\left(e+l\right)g} \end{array}\right)=\left(\begin{array}{cccc} b_{11} & b_{12} & \cdots & b_{1g}\\ \vdots & \vdots & \; & \vdots \\ b_{r1} & b_{r2} & \cdots & b_{rg} \end{array}\right), \end{array}$$

where *P*_*ijk*_ is a collection of the *k*th multisource traffic information obtained by various collection devices on the *j* section; *A* is the matrix formed by *a*_*ji*_; *b*_*jk*_ is the *k*th multi-source traffic information obtained on the *j* section; *g* for multisource traffic information collection = the number of equipment categories, then

(18) $$\begin{array}{c} P_{ijk}=\left(\begin{array}{cccc} b_{11}^{\prime } & b_{12}^{\prime } & \cdots & b_{1g}^{\prime }\\ \vdots & \vdots & \; & \vdots \\ b_{r1}^{\prime } & b_{r2}^{\prime } & \cdots & b_{rg}^{\prime } \end{array}\right). \end{array}$$

The effect maximization function of multisource traffic information collection equipment is

(19) $$\begin{array}{c} \max U=\sum_{j=1}^{r}\sum_{k=1}^{g}b_{jk}^{\prime }f_{j}r_{j}, \end{array}$$

where *b*_*jk*_′ is the *k* multisource traffic information jointly acquired by all collection equipment on the *j* section, when *b*_*jk*_′ ≥ 1, *b*_*jk*_′=1, otherwise *b*_*jk*_′=0,. The coverage rate of mobile multisource traffic information collection equipment *y* is

(20) $$\begin{array}{c} \gamma_{y}=\frac{\sum_{j=1}^{r}a_{yj}}{r}, \end{array}$$

where *a*_*yj*_ as the coverage rate of the Y multisource traffic information collection equipment in section *j*.

Finally, the sample supply of *y* is clarified. In addition, on the basis of benefit maximization and economic benefit optimization, the planning function of multiple objectives is

(21) $$\begin{array}{c} \left\{\begin{array}{c} \min Z=\sum_{i=1}^{l}\sum_{j=1}^{r}a_{ji}\times \varphi_{ji}\times c_{i}+\sum_{i=1}^{e}n_{i}c_{i}\\ \max U=\sum_{i=1}^{l}\sum_{j=1}^{r}b_{jk}f_{j}r_{j}\\ \text{s.t. }\left\{\begin{array}{c} a_{ji}\geq 0,\;a_{ji}=0\text{ or }1\\ b_{jk}=\sum_{i=1}^{l+e}a_{ji}\times x_{ik}\\ b_{jk}^{\prime },\text{ if }b_{jk}\geq 1,\;b_{jk}^{\prime }=1,\text{ otherwise},\;b_{jk}^{\prime }=0\\ n_{y}=\phi \left(\sum_{j=1}^{r}a_{yj}\right) \end{array}\right. \end{array}\right.. \end{array}$$

#### 3.3.3. Solving Algorithm

In order to solve the multiobjective planning function problem, the first step is to maximize the function

(22) $$\begin{array}{c} \left\{\begin{array}{c} \min Z=-\sum_{i=1}^{l}\sum_{j=1}^{r}a_{ji}\times \varphi_{ji}\times c_{i}-\sum_{i=1}^{e}n_{i}c_{i}\\ \max U=\sum_{i=1}^{l}\sum_{j=1}^{r}b_{jk}^{\prime }f_{j}r_{j}\\ \text{s.t. }\left\{\begin{array}{c} a_{ji}\geq 0,\;a_{ji}=0\text{ or }1\\ b_{jk}=\sum_{i=1}^{l+e}a_{ji}\times x_{ij}\\ b_{jk}^{\prime },\text{ if }b_{jk}\geq 1,\;b_{jk}^{\prime }=1,\text{ otherwise},\;b_{jk}^{\prime }=0\\ n_{y}=\phi \left(\sum_{j=1}^{r}a_{yj}\right) \end{array}\right. \end{array}\right.. \end{array}$$

During the process of solving, due to existence of certain conflicts between multiple targets, it is difficult to obtain the ideal optimal solution, so intelligent algorithms can be used to obtain the optimal solution.

In this paper, we use a multisfunctional planning solution based on genetic algorithm to calculate [11]. First, the priority solution *f*(*x*^*∗*^) is determined, and the solution is 337519; 39 can be found around the solution. (*X*^*∗*^) is the satisfaction solution of the model.

By checking the correlation between the two, two gray correlations can be expressed, that is, a single objective function converted into a solution

(23) $$\begin{array}{c} \left\{\begin{array}{c} \max\nabla \left(f,f^{\prime }\right)\\ s.t.\left\{\begin{array}{c} a_{ji}\geq 0,a_{ji}=0\text{ or }1\\ b_{jk}=\sum_{i=1}^{l+e}a_{ji}\times x_{ik}\\ b_{jk}^{\prime },\text{ if }b_{jk}\geq 1,\;b_{jk}^{\prime }=1,\text{ otherwise},\;b_{jk}^{\prime }=0\\ n_{y}=\phi \left(\sum_{j=1}^{r}a_{yj}\right) \end{array}\right. \end{array}\right., \end{array}$$

where the gray correlation ∇(*f*, *f*′) is

(24) $$\begin{array}{c} \nabla \left(f,f^{\prime }\right)=\frac{\min\left|f^{\prime }\left(x^{*}\right)-f\left(x^{*}\right)\right|+\xi \max\left|f^{\prime }\left(x^{*}\right)-f\left(x^{*}\right)\right|}{\left|f^{\prime }\left(x^{*}\right)-f\left(x^{*}\right)\right|+\xi \max\left|f^{\prime }\left(x^{*}\right)-f\left(x^{*}\right)\right|}. \end{array}$$

Assuming there are *p* objective functions in total, the solution model based on the gray relational degree algorithm is

(25) $$\begin{array}{c} \left\{\begin{array}{c} \max\overset{k}{\underset{p=1}{\sum }}\omega_{p}\frac{\min\left|f^{\prime }\left(x^{*}\right)-f\left(x^{*}\right)\right|+\xi \max\left|f^{\prime }\left(x^{*}\right)-f\left(x^{*}\right)\right|}{\left|f^{\prime }\left(x^{*}\right)-f\left(x^{*}\right)\right|+\xi \max\left|f^{\prime }\left(x^{*}\right)-f\left(x^{*}\right)\right|}\\ \text{s.t. }\left\{\begin{array}{c} a_{ji}\geq 0,\;a_{ji}=0\text{ or }1\\ n_{y}=\phi \left(\overset{r}{\underset{j=1}{\sum }}a_{yj}\right) \end{array}\right. \end{array}\right.. \end{array}$$

The approximate optimal solution is obtained through genetic algorithm, and the final optimal solution is obtained through repetition.

### 3.4. Parking Lot Location Guidance Algorithm Based on Dijkstra Algorithm

Generally speaking, Dijkstra's algorithm uses an adjacency matrix for storage. The adjacency matrix stores the relationship between each node. However, considering the actual situation of urban roads, although there are many nodes in the road graph, there are few directly connected points. Therefore, the actual road graph is a sparse graph, and the corresponding adjacency matrix is also a sparse matrix. Therefore, if the adjacency matrix is used to store the nodes, a lot of space will be wasted.

In this paper, the adjacency list is used to replace the adjacency matrix, which can reduce the space complexity of the algorithm. The comparison of the two storage methods is as follows:

If it is assumed that there are *b* nodes in the graph *G*=(*V*, *E*), then its adjacency matrix A is a symmetric matrix.

If *G* is an unweighted graph, its adjacency matrix can be expressed as

(26) $$\begin{array}{c} A\left[i,j\right]=\left\{\begin{array}{cc} 1, & \left(V_{i},V_{j}\right)\in E\left(G\right),\\ 0, & \left(V_{i},V_{j}\right)\notin E\left(G\right). \end{array}\right. \end{array}$$

We believe that the actual city road map is an undirected weighted map. In the adjacency matrix of *G*=(*V*, *E*), the elements are

(27) $$\begin{array}{c} A\left[i,j\right]=\left\{\begin{array}{cc} \omega_{ij}, & \left(V_{i},V_{j}\right)\in E\left(G\right),\\ 0, & \left(V_{i},V_{j}\right)\notin E\left(G\right)\text{ \& }i=j,\\ \infty , & \left(V_{i},V_{j}\right)\notin E\left(G\right)\text{ \& }i\neq j. \end{array}\right. \end{array}$$

**Figure 5:**
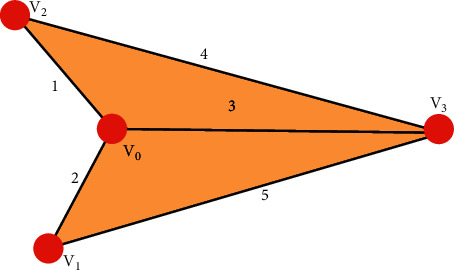
A typical undirected weighted graph.

That is, the element on the symmetry line of the adjacency matrix is 0, where *ω*_*ij*_ is the weight between two points, and *∞* represents that *i* and *j* are not connected. A typical undirected weighted graph is shown in Figure 5:

The adjacency matrix is

(28) $$\begin{array}{c} A=\left[\begin{array}{cccc} 0 & 1 & 2 & 3\\ 1 & 0 & \infty & 4\\ 2 & \infty & 0 & 5\\ 3 & 4 & 5 & 0 \end{array}\right]. \end{array}$$

On the one hand, the storage space of the adjacency matrix is only related to the number of nodes and contains *n*^2^ elements in total. However, since the adjacency matrix is mostly a sparse graph, a lot of space is wasted. On the other hand, the time complexity of the Dijkstra algorithm using the adjacency matrix storage method is *O*(*n*^2^), and the time cost is too large.

**Figure 6:**
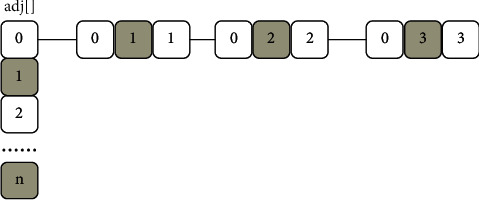
*v*_o_ example diagram.

The adjacency list is a very useful storage structure for sparse graphs. All the nodes in the graph are stored in a one-dimensional array, and then the edge with it as the end point is linked after each node to form a linked list. The linked list node is composed of three parts: the start point, the end point, and the weight of the edge. As shown in Figure 6, *v*_0_ is a three-way intersection, and there are 3 sides with it as the endpoint. Then, we can build a singly linked list connecting these 3 edges and then connect it to *v*_0_.

**Figure 7:**
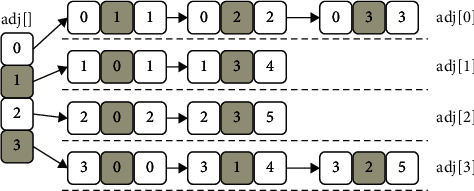
Adjacency list of undirected weighted graph.

Figure 7 is stored as an adjacency list as follows:

The adjacency table storage requires only *n* + *m* storage units, far less than *n*^2^.

From the perspective of space complexity, the adjacency table only needs *n*+2*m* storage units, while the adjacency matrix requires *n*^2^, and the storage efficiency advantage of the adjacency table is significant. In terms of time complexity, the complexity of the adjacency list *O*(*m*+*n*) is also much lower than the complexity of the adjacency matrix *O*(*n*^2^).

In the research process, the graph is regarded as a plane graph, and the shortest path value from the temporarily marked node to the starting node and the Euclidean distance to the target node are added, and this value is regarded as the characteristic value of the marked node. In the selection process of permanent marked nodes, it is necessary to use this value as a theoretical basis to select among the set of temporary marked nodes, and permanent marked nodes can select the smallest temporary node of this value. This method can ensure that the target node can be found more accurately and quickly through the Dijkstra algorithm, and the search efficiency can be improved.

**Figure 8:**
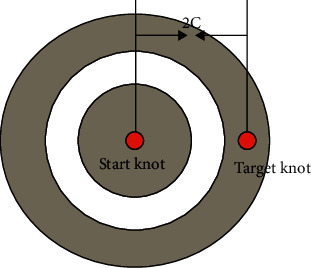
Classic Dijkstra algorithm.

**Figure 9:**
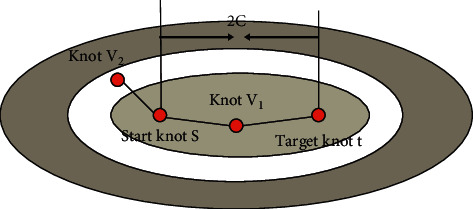
The optimized Dijkstra algorithm.

**Figure 10:**
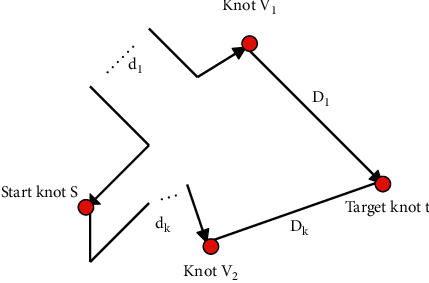
Schematic diagram of optimized Dijkstra algorithm.

Through the above algorithm, the premise of determining the permanent mark point is that the above two values add to the minimum value. The concept of an ellipse here is a trajectory corresponding to a point in the surface where the distance between *s* and *t* is added to a constant 2c. Among them, the point *p* is located on the trajectory, and the distances to the two points *s* and *v* are *d*_*sp*_ and *d*_*tp*_, respectively, then *d*_*sp*_+*d*_*tp*_=2*c* can be obtained. Through this traversal method to find the shortest path method, the end point and the meta point can be regarded as two focal points, which can be regarded as a concentric ellipse. Before Dijkstra algorithm is optimized, the corresponding search process is shown in Figure 8:

After Dijkstra algorithm is optimized, the corresponding search process is shown in Figure 9:

If nodes *v*_1_ and *v*_*k*_ are both temporarily marked nodes, *s* is the starting node, and *t* is the target node. It can be seen from Figure 10 that *d*_1_ and *d*_*k*_ are the shortest path lengths between *v*_1_ and *v*_*k*_ and *s*, respectively, and *D*_1_ and *D*_*k*_ are the Euclidean distances between *v*_1_ and *v*_*k*_ and *t*, respectively. The Euclidean distance between *v*_1_ and *v*_*k*_ is *C*.

In the Dijkstra algorithm before optimization, in terms of selecting marked nodes, if *d*_1_ > *d*_*k*_, the marked node selects *v*_*k*_, otherwise it is *v*_1_. After the algorithm is optimized, if *d*_1_+*D*_1_ > *d*_*k*_+*D*_*k*_, then mark the node selects *v*_*k*_, otherwise it is *v*_1_.

In summary, the search range of the latest algorithm is an ellipse. After optimization, the search efficiency is improved and the number of traversed nodes is reduced.

**Figure 11:**
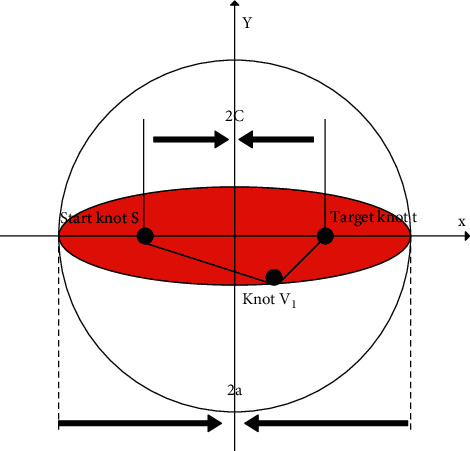
Search range restricted by ellipse.

The ellipse limits the range of the search area, see Figure 11 for details. *s* is the starting node, *v*_1_ is the boundary node, *t* is the target node, and 2*a* is the maximum limit distance. The Euclidean distance between *s* and *v*_1_ is denoted by |*sv*_1_|, and the Euclidean distance between *v*_1_ and *t* is denoted by |*v*_1_*t*|, and the specific search range is |*sv*_1_|+|*v*_1_*t*| < 2*a*. If |*sv*_1_|+|*v*_1_*t*| > 2*a*, then these nodes can be ignored in the algorithm. The reason is that the distance traveled by *s* from *v*_1_ to *t* is greater than 2*a*. In this way, an ellipse is formed, the ellipse is composed of all nodes that meet the condition of |*sv*_1_|+|*v*_1_*t*|=2*a*, the focus is *s* and *t*, and the major axis is 2*a*.

In summary, the parameters are set as follows: *s* and *t* are selected for the ellipse focus, and *λ|st|* is selected for the length of the major axis. The Euclidean distance between *s* and *t* is represented by |*st*|, and the confidence coefficient is *λ*.

In the process of setting parameters, the main purpose is to find a more reasonable long axis, that is, to calculate the most reasonable *λ*, and get the desired ellipse search interval.

The principle of finding *λ*: among the passing road network nodes, a group of nodes with strong representativeness is selected, and the starting node set and the target node are formed by multiplying A and B, which is the C composed of A and B of the required shortest path. C is also a set, that is,

(29) $$\begin{array}{c} C=A\times B\\ =\left\{\left(a,b\right)|aEA\alpha beB\right\}. \end{array}$$

Among them, the starting node is represented by *a*_*l*_, the target node is represented by *b*_*l*_, the Euclidean distance between the two is represented by *E*_*a*_*l*_*b*_*l*__, and *P*_*a*_*l*_*b*_*l*__ is the specific value of the shortest path. Through *λ*_*i*_=*P*_*a*_*l*_*b*_*l*__/*E*_*a*_*l*_*b*_*l*__, *λ*_*i*_ can be obtained. Regarding what kind of data is selected, the coefficient size of the specific ratio is obtained, and it is expressed in the form of a set, which is Γ. By studying the elements in it, *λ*_0_ is obtained. Then, at *λ*_*i*_ ≤ *λ*_0_, the confidence level is 95%. Then, *λ*_0_ is the specific coefficient size, so the size of the major axis can be obtained: 2*a*=*λ*_0_|*st*|.

**Figure 12:**
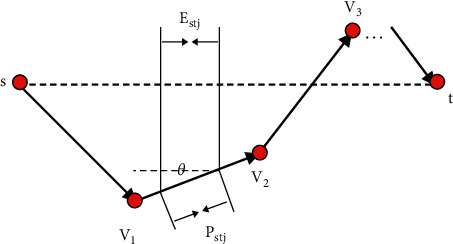
Schematic diagram of the research on the rule of *λ* value.

It can be seen from Figure 12 that *s* belongs to any element in A, *t* belongs to any element in B, *λ*=*P*_*st*_/*E*_*st*_, and the shortest path between *s* and *t* is represented by *P*_*st*_, which is *s*⟶*v*_1_⟶*v*_2_⟶*v*_3_⟶⋯⟶*t*. The Euclidean distance between *s* and *t* is denoted by *E*_*st*_. In the process of calculating *λ*, *E*_*st*_ is divided into segments, the number is *n*, and any segment is denoted as *E*_*st*1_=*E*_*st*2_=⋯=*E*_*st*_=*E*_*stn*_(*i*=1,2,3,…, *n*). According to the situation of segmentation, *P*_*st*_ is also segmented, the number is also *n*, and any segment is *P*_*st*1_=*P*_*st*2_=⋯=*P*_*sti*_=*P*_*stn*_(*i*=1,2,3,…, *n*), and there is *P*_*sti*_=*E*_*sti*_/cos  *θ*_*i*_.

The calculation process of *λ* is

(30) $$\begin{array}{c} \lambda =\frac{P_{st}}{E_{st}}\\ \;=\frac{P_{st1}+P_{st2}+\cdots +P_{sti}+\cdots +P_{stn}}{E_{st}}\\ \;=\frac{1}{n}\left(\frac{1}{\cos\;\theta_{1}}+\frac{1}{\cos\;\theta_{2}}+\cdots +\frac{1}{\cos\;\theta_{n}}\right)\\ \;=\frac{1}{n}\sum_{i=1}^{n}\frac{1}{\cos\;\theta_{i}}. \end{array}$$

In urban roads, *θ*_*i*_ generally takes a value on [0, *π*/4]. The following three cases discuss the value of *λ*:

(1) If *s* and *t* have a relatively close distance, *n* has a smaller value. In order to prevent the shortest path from being found in the search range and to ensure that all valid nodes are included in the ellipse range, the maximum value of *λ* can be selected at this time.
  (31) $$\begin{array}{c} \lambda_{\max}=\frac{1}{n}\sum_{i=1}^{n}\frac{1}{\cos\;\theta_{i}}\\ =\frac{1}{n}\cdot n\sqrt{2}\\ =\sqrt{2}. \end{array}$$
(2) If *s* and *t* have a very long distance, *n* is infinite. On [0, *π*/4], *θ*_*i*_ conforms to a uniform distribution, then *λ* is
  (32) $$\begin{array}{c} \lambda_{\infty }=\int_{0}^{\frac{\pi }{4}}\frac{1}{\cos\;\theta }\frac{4}{\pi }\text{d}\theta \\ ={\frac{4}{\pi }\ln\left|\sec\;\theta +\tan\;\theta \right||}_{0}^{\frac{\pi }{4}}\\ =\frac{4}{\pi }\ln\left(\sqrt{2}+1\right)\\ \approx 1.122. \end{array}$$
(3) If *s* and *t* have a longer distance, *n* has a larger value, and *λ* basically conforms to the normal distribution, then *N*(*μ*, *σ*^2^/*n*), and the specific formula is

(33) $$\begin{array}{c} \mu =E\left(\frac{1}{\cos\;\theta }\right)\\ =\int_{0}^{\frac{\pi }{4}}\frac{1}{\cos\;\theta }\frac{4}{\pi }\text{d}\theta \\ \approx 1.122,\\ \sigma^{2}=D\left(\frac{1}{\cos\;\theta }\right)\\ =E\left(\frac{1}{\cos^{2}\;\theta }\right)-{\left[E\left(\frac{1}{\cos\;\theta }\right)\right]}^{2}\\ =\int_{0}^{\frac{\pi }{4}}\sec^{2}\theta \frac{4}{\pi }\text{d}\theta -{\left[\int_{0}^{\frac{\pi }{4}}\frac{1}{\cos\;\theta }\frac{4}{\pi }\text{d}\theta \right]}^{2}\\ =\frac{4}{\pi }-{\left[\frac{4}{\pi }\ln\left(\sqrt{2}+1\right)\right]}^{2}\\ \approx 0.01412. \end{array}$$

It can be seen that the larger the value of *n*, the more normal distribution is obeyed. Therefore, in order to obtain more accurate data results, this paper chooses the ellipse with the largest area, then *λ* = 1.414. Moreover, when there is a long distance between *s* and *t*, when the study is conducted from the perspective of normal distribution, according to the confidence level query, the confidence level reaches 95%.

The performance analysis of the ellipse search algorithm is as follows:

(1) Algorithm validity analysis
In this regard: the main focus of attention is running time. The search range of the Dijkstra algorithm before optimization is
  (34) $$\begin{array}{c} S_{\text{Diksrva}}=\pi r^{2}\\ =\pi {\left(\frac{\lambda +1}{2}\left|st\right|\right)}^{2}. \end{array}$$
The search range of the ellipse restricted area algorithm is
  (35) $$\begin{array}{c} S_{\text{llipse}}=\pi ab\\ =\pi \frac{\left|st\right|}{2}\frac{\sqrt{\left(x^{2}-1\right)\left|st\right|}}{2}. \end{array}$$
(2) Reliability analysis of the algorithm
The so-called reliability is to ensure that the path found belongs to the shortest path.
In a specific road network, nodes do not exist alone and have strong connectivity. There are basically paths connected between two points. If there is a small straight-line distance between the starting node and the target node, it will have a relatively small distance. The traversal range is small, and the path to China Unicom may not be found. In order to avoid this problem, in the research process of this paper, the eccentricity selected by the ellipse algorithm is the largest, that is, the ellipse area is the largest.
In summary, in the case of *λ* = 1.414, the confidence level is 95%, that is, only 5% may not be able to find the required shortest path.
(3) Algorithm applicability analysis

In the application process of the ellipse search algorithm, the search range is limited, the work efficiency is improved, and the traversal time is reduced. However, in order to make it clear that all newly appearing nodes are within this range through this method, it is necessary to perform many operations of product and square root extraction, so it takes a lot of time.

The algorithm flow is

The first step is to initialize the data. The algorithm clarifies *s* and *t* and calculates the straight-line distance |st| between them based on their latitude and longitude calculations.

The second step is to define the ellipse area. The focus is *s* and *t*,  *λ*=1.414, from which the eccentricity can be known, and the elliptical area can be obtained.

The third step is to select the breadth-first search method to find the node and compare the value of the sum of the Euclidean distance between the node to *b* and *t* with the length of the long axis of the ellipse. If |*sv*_1_|+|*v*_1_*t*| < 2*a*, then this node is included in the area.

The fourth step is to find the shortest path st in the ellipse interval using Dijkstra's algorithm and then terminate the algorithm.

Through the understanding of the algorithm of the ellipse restricted search area, it is found that the calculation process has a slower speed, takes more time, and has lower work efficiency. Therefore, the algorithm is analyzed and researched. In the calculation process of the rectangular restricted search area algorithm, the required result can be obtained without a lot of calculation, and it has a good effect. The algorithm mainly calculates the circumscribed rectangle of the ellipse area and treats it as a restricted search area, so it achieves the effect of reducing the calculation amount of the algorithm.

**Figure 13:**
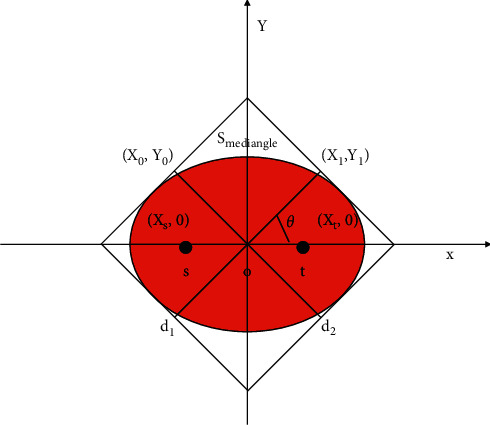
Schematic diagram of ellipse algorithm coordinates.

The specific content is shown in Figure 13. *s* is the starting node, *t* is the target node, and the ellipse equation can be described by *s*, *t*, *λ*. In this process, in order to make the calculation easier, the center of the ellipse is assumed to be the origin of the coordinates, and the two focal points of the ellipse are above the *g* axis, then the coordinates (−*x*_*s*_, 0) of *s* and the coordinates (*x*_*t*_, 0) of *m* are obtained, and *x*_e_=*x*_*t*_=*c*.

The elliptic equation is

(36) $$\begin{array}{c} \frac{x^{2}}{a}+\frac{y^{2}}{b}=1. \end{array}$$

The parametric equation is

(37) $$\begin{array}{c} \left\{\begin{array}{c} x=a\;\cos\;\theta \\ y=b\;\sin\;\theta \end{array}\left(\theta \in \left[0,2\pi \right]\right)\right.. \end{array}$$

In the above formula, *c*^2^+*b*^2^=*a*^2^, *a*=*λc*.

The elliptic equation is used to derive the derivative of x:

(38) $$\begin{array}{c} y^{\prime }=-\frac{b^{2}}{a^{2}}\frac{x}{y}. \end{array}$$

As shown in Figure 13, when a point (*x*_0_, *y*_0_) is taken, the tangent equation is

(39) $$\begin{array}{c} y=-\frac{b^{2}}{a^{2}}\frac{x}{y}\left(x-x_{0}\right)+y_{0}. \end{array}$$

When *m*=−*b*^2^/*a*^2^*x*_0_/*y*_0_, the tangent equation is

(40) $$\begin{array}{c} y=mx\pm \sqrt{a^{2}m^{2}+b^{2}}. \end{array}$$

Then the distance between the two tangents is

(41) $$\begin{array}{c} d_{1}=\frac{2\sqrt{a^{2}m^{2}+b^{2}}}{\sqrt{m^{2}+1}}. \end{array}$$

**Figure 14:**
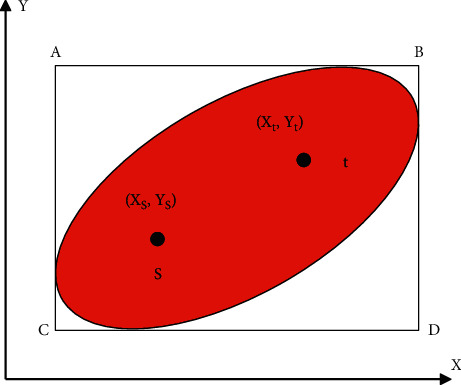
Comparison of ellipse and rectangular restricted areas.

As shown in Figure 14, we take a point (*x*_1_, *y*_1_) and its tangent is

(42) $$\begin{array}{c} y=-\frac{1}{m}x\pm \sqrt{\frac{a^{2}}{m^{2}}+b^{2}}. \end{array}$$

Then the distance between the two tangents is

(43) $$\begin{array}{c} d_{2}=\frac{2\sqrt{a^{2}+b^{2}m^{2}}}{\sqrt{m^{2}+1}}. \end{array}$$

If the ellipse has a circumscribed rectangle with the smallest area, then the formulas (38) and (40) can be used to obtain *S*_rectangle _:

(44) $$\begin{array}{c} S_{\text{rectangle }}=d_{1}\cdot d_{2}=\frac{4\sqrt{a^{2}m^{2}+b^{2}}\cdot \sqrt{a^{2}+b^{2}m^{2}}}{m^{2}+1}. \end{array}$$

It can be seen from the above formula that if *m*^2^ is equal to 0, that is, in the case of *m*=0, the ellipse has a circumscribed rectangle with the smallest area. If the tangent line passes through (*x*_0_, *y*_0_), it is parallel to the axis, and if the tangent line passes through (*x*_1_, *y*_1_), it is parallel to the axis.

If *m*=0, that is, in the case of *θ*=*kπ*/2(*k*=0,1,2,3,…), the area of the rectangle is the minimum:

(45) $$\begin{array}{c} S_{\text{rectargle }}=4ab=4\lambda \sqrt{\lambda^{2}-1}{\left|st\right|}^{2}. \end{array}$$

If *m*=1, that is, in the case of *θ*=*π*+2*kπ*/2(*k*=0,1,2,3,…), the area of the rectangle is the maximum:

(46) $$\begin{array}{c} S_{\text{rectargle }}=2\left(a^{2}+b^{2}\right)=2\left(2\lambda^{2}-1\right){\left|st\right|}^{2}. \end{array}$$

It can be seen from Figure 14 that the figure can be obtained as a standard ellipse rotation. The rotation angle is *θ*, and it translates *x*_*c*_ and *y*_*c*_ in the *x* and *y* directions, respectively. The elliptic equation can be obtained by the coordinate values of *s* and *t*:

(47) $$\begin{array}{c} \frac{{\left[\left(x-x_{c}\right)\cos\;\theta +\left(y-y_{c}\right)\sin\;\theta \right]}^{2}}{a^{2}}+\frac{{\left[-\left(x-x_{c}\right)\sin\;\theta +\left(y-y_{c}\right)\cos\;\theta \right]}^{2}}{b^{2}}=1. \end{array}$$

In the above formula, *c*^2^+*b*^2^=*a*^2^,  *x*_*c*_=*x*_*s*_+*x*_*t*_/2,  *y*_*c*_=*y*_*s*_+*y*_*t*_/2,  *θ*=arctan*y*_*t*_ − *y*_*s*_/*x*_*t*_ − *x*_*s*_. *x* and *y*:

(48) $$\begin{array}{c} x_{m}=x_{c}\pm \sqrt{a^{2}\cos^{2}\;\theta +b^{2}\sin^{2}\;\theta },\\ y_{m}=y_{c}\pm \sqrt{b^{2}\cos^{2}\;\theta +a^{2}\sin^{2}\;\theta }. \end{array}$$

By reduction, the boundary of the rectangular area is obtained:

(49) $$\begin{array}{c} x_{m}=x_{c}\pm \left|st\right|\sqrt{\lambda^{2}-\;\sin^{2}\;\theta },\\ y_{m}=y_{c}\pm \left|st\right|\sqrt{\lambda^{2}-\;\cos^{2}\;\theta }. \end{array}$$

Then, the area of the rectangular area is *S*_rectargle _ ∈ [4*ab*, 2(*a*^2^+*b*^2^)]

Performance analysis of rectangle search algorithm:

#### 3.4.1. Algorithm Validity Analysis

If the area of the rectangle is the smallest, the ratio of the search area of the rectangle and the ellipse is

(50) $$\begin{array}{c} \frac{S_{\text{rectargle }}}{S_{\text{ellipse }}}=\frac{4ab}{\pi ab}\approx 1.27. \end{array}$$

If the area of the rectangle is the largest, the ratio of the search area of the rectangle and the ellipse is

(51) $$\begin{array}{c} \frac{S_{\text{rectangle }}}{S_{\text{ellipse }}}=\frac{2\left(a^{2}+b^{2}\right)}{\pi ab}\\ =\frac{4\lambda^{2}-2}{\pi \lambda \sqrt{\lambda^{2}-1}}. \end{array}$$

### 3.5. Parking Space Reservation Module

**Figure 15:**
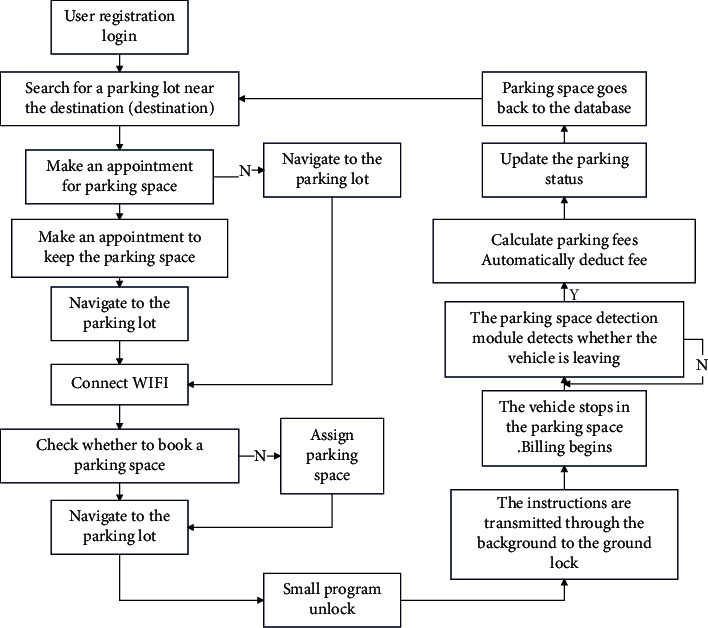
Business process of parking space reservation.

After the user finds the target parking lot, he may need to make a parking space reservation. Generally speaking, there are two ways to make a reservation: reserve a parking lot or reserve a parking space. The following is an analysis of possible reservation methods: (1) reserve a parking lot with precise reservation: if the reservation method provided to the parking lot of the parking lot means that it cannot fully guarantee that the user will leave a space for reserved vehicles after driving to the parking lot. Therefore, scientific traffic forecasting is required to ensure that a certain number of reserved vehicles arrive at the time of arrival. It can also guarantee a certain parking space. The calculation process of this method is too complicated, and for the peak parking period, the supply is often in short supply. Therefore, it is difficult to realize the reservation behavior of reserved parking. Fuzzy reservation: the parker makes a reservation for a fixed parking time. The parking lot guarantees that the parking space is free at the scheduled time and will not be illegally occupied. In order to ensure the above effects, it is necessary to ensure that there is a certain amount of vacant parking spaces that can be turned over. The berth cannot be used at the highest efficiency. (2) Precise reservation of the reserved parking space: the parking space and the start and end time are accurately reserved by the parker. The system needs to have parking space status detection equipment and parking space occupancy equipment. But for CBD commercial buildings, hospitals, and other areas, parking behavior is of high frequency and changeable. A parking person may delay parking and affect the next scheduled parking person to park the vehicle. Therefore, this kind of contradiction is difficult to solve, so stop. Accurate prediction of the position is not feasible. Fuzzy reservation + precise reservation: fuzzy means that you do not book an accurate parking start time, and precision means that the object of pleasure is the parking space. When a parker chooses a predetermined behavior, the general understanding is that the parker is worried that it is difficult to find a parking space within the arrival time or that the parking space best meets the user's expectations and wants to book in advance. Therefore, the system adopts this method. Specifically, the user has reserved a parking space in a parking lot, and since the reservation, the user's billing starts. In order to balance the user's affordability and the parking revenue, the reservation fee is set as 50% parking fee, the strategy can be optimized in the future, such as different charging standards in different time periods. In summary, only the fuzzy reservation + precise reservation mode for parking spaces is suitable for this system. Parkers can decide whether to reserve a parking space based on the predicted number of empty parking spaces mentioned in the previous chapter and their own needs. If there is a reservation in this mode, the reservation function can be designed with reference to the reservation business process, as shown in Figure 15:

When the user clicks on a parking lot in the reservation module, he enters the parking space reservation details page. The module has a parking space map of the parking lot, and the parking space map indicates the best parking space recommended by the system. The most important factor in the problem of the best parking space in the parking lot is the parking distance, which can be regarded as the shortest path problem (other factors have a weak influence and are not considered for the time being). That is to find the minimum value of stopping distance + driving distance away, so the shortest path algorithm is used to calculate the parking space and guidance path of the shortest path. This module can greatly improve the decision-making efficiency of parking seekers.

Suppose the entrance of the parking lot is *E* and the exit is *S*. If a certain free parking space is *Pi* (*i* = 1,2,…, *n*), the shortest path corresponding to the entrance process is path(*E*, *P*), and the shortest path corresponding to the exit process is path(*p*_*i*_, *s*).

Then the entire shortest path length corresponding to the parking space is *d*_path_(*E*, *p*_*i*_)+path(*p*_*i*_, *s*)

The shortest path length corresponding to the best parking space can be described asmin{*d*_path_(*E*, *p*_1_)+path(*p*_*i*_, *s*), *d*_path_(*E*, *p*_2_)+path(*p*_2_, *s*),…, *d*_path_(*E*, *p*_*n*_)+path(*p*_*n*_, *s*)}.

In other words, the core of this algorithm is to find the point and path of the minimum value of *d*_path_(*E*, *p*_*i*_)+path(*p*_*i*_, *s*).

## 4. Development and Design of System Software

**Figure 16:**
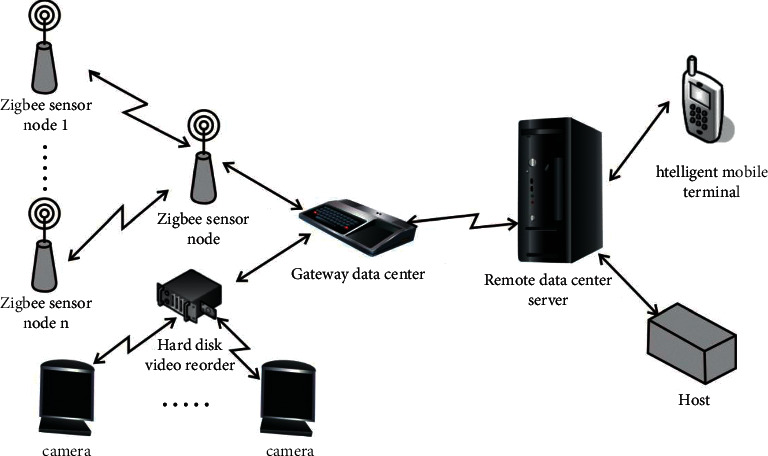
System network networking design.

Based on the design of the above-mentioned multisource traffic information collection and combination algorithm, the intelligent multisource traffic information collection combination optimization model is designed for the network design and software design of the multisource traffic information collection combination optimization model, to carry out the network design of multisource traffic information collection combination optimization mode. ZigBee network technology is used to build a three-layer structure model of multisource traffic information collection and optimization model, and ITU-TH.323 and IETFSIP network signaling is used for multisource traffic information collection combination optimization model network control, to design human-computer interaction module, to realize multisource traffic information collection combination optimization mode human-computer interaction response design, an to build in a single network environment multisource traffic information fusion and information dispatch center (Figure 16).

In the MVB bus control protocol and embedded environment, the software development and design of the multisource traffic information collection combination optimization model is realized, and the ZigBee, GPRS, and other network technologies are used to carry out the network design of the multisource traffic information platform. Under the B/S structure system, the network design of the multisource traffic information collection combination optimization model is carried out, the process management and bus scheduling are carried out under the MVB bus control protocol, the Sip protocol stack is used to establish the session protocol of the multisource traffic information platform, and SIP is called. The call interface is used to create an INVITE message, and the data source is wrote to the multisource traffic information collection and combination optimization model DataSet. during Web programming, ADO. NET is used to query, update, and database management of the large-scale multisource traffic information collection and optimization model. Through the above analysis, the software development and design of the system can be realized.

**Figure 17:**
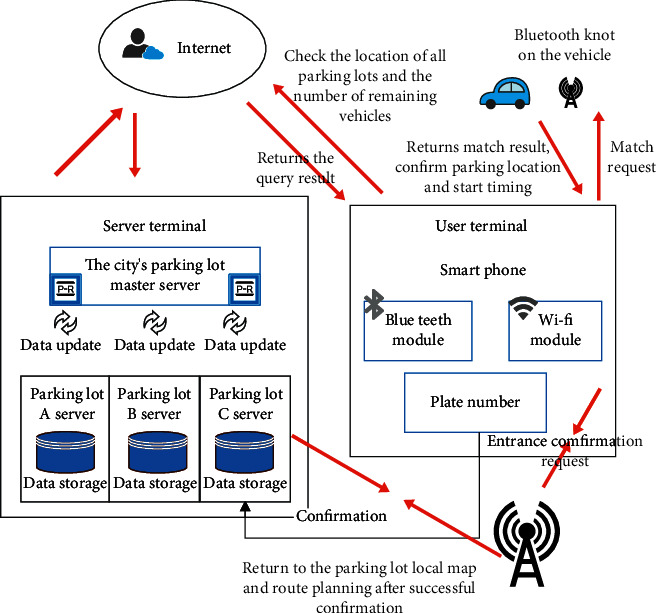
Overall system architecture.

The intelligent parking lot management system designed in this paper is mainly composed of parking lot server and mobile phone client. Communication methods include network communication based on TCP/IP protocol, Bluetooth communication, and Wi-Fi communication. The server uses a PC with Window 10 operating system as the socket communication and data storage device, responsible for socket communication with the mobile phone, and manages user information and parking lot information; adopts the ARM 6410-based development board as the core control of Bluetooth communication module, expand the Bluetooth module outwards, responsible for Bluetooth communication with mobile phones; use Wi-Fi routers to establish a local area network to simulate network communication between users and servers; and simulate the scene of opening the door when users arrive at the entrance of the parking lot. The mobile client uses a smartphone with Android operating system, equipped with Wi-Fi function, Bluetooth function, and GPS positioning function, etc. The Wi-Fi function is used to detect Wi-Fi signals and communication within the local area network. The Bluetooth function is used to communicate with Bluetooth nodes in the parking space. GPS positioning is used to query the current position. The SDK provided by Baidu Maps gives the distance from the current position to the parking lot. The SDK provided by Baidu Maps gives the driving route from the current location to the entrance of the parking lot. The overall architecture of the intelligent parking lot guidance system designed in this paper is shown in Figure 17.

**Figure 18:**
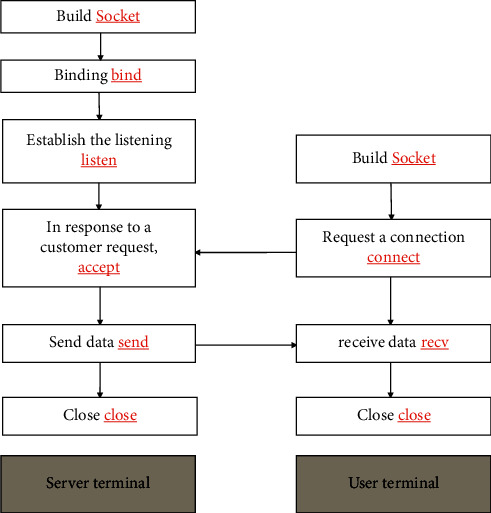
TCP/IP protocol workflow.

In order to prevent data loss during the communication process between the user and the server, this design adopts a connection-oriented and reliable TCP/IP protocol, and the communication process is implemented through the socket interface. The function of socket is mainly to provide an interface for data transmission between two different programs. Users only need to know the IP address and port of the server to establish a connection with the server and perform network communication. The workflow is shown in Figure 18.

**Figure 19:**
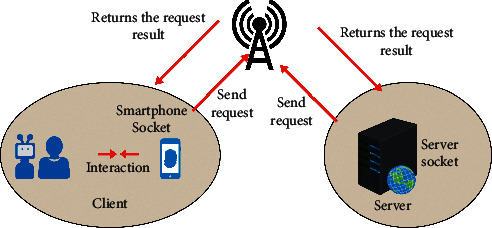
Schematic diagram of client accessing server.

In this design, the user's mobile phone first accesses the LAN Wi-Fi and automatically obtains an IP address. Establish a connection with the server through the server's IP address and port. The establishment of each connection requires the server to open a new socket thread to receive the registration, login, query, appointment, and other requests sent by the client to the server after the server receives the request, Through the operation of inserting and modifying the database, realize the functions of user registration and appointment; through the query operation to verify the correctness of the user information. The server returns the request result to the user through the user's IP address, as shown in Figure 19.

**Figure 20:**
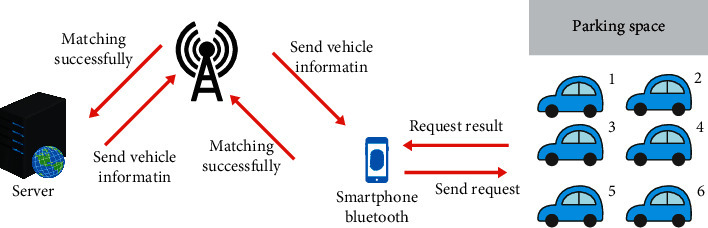
System Bluetooth composition block diagram.

In this design, an independent Bluetooth node is installed in each parking space, and the Bluetooth node and the parking number are bound one-to-one and stored in the database. When the user receives the parking space address from the server, the Bluetooth on the mobile phone is automatically turned on and starts to detect the Bluetooth address on the parking space. Since the distance of Bluetooth communication is only within 10 m, when the user's car reaches the parking space, the pressure sensor detects the pressure signal. After turning on the Bluetooth module, the mobile phone Bluetooth can search for the address of the Bluetooth module, which can ensure that the user will park the car in the correct parking space and will not match the Bluetooth in other parking spaces. The Bluetooth matching process adopts an automatic matching method. Automatic matching requires program control on the mobile phone. This can reduce the user's operation on the mobile phone and improve the safety of the user's driving. The Bluetooth composition block diagram of this system is shown as in Figure 20.

**Figure 21:**
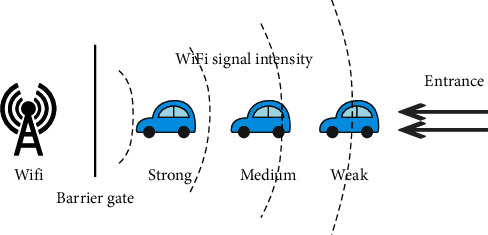
Wi-Fi signal diagram at the entrance of the parking lot.

Figure 21 is a schematic diagram of the entrance to the parking lot. According to the data, there is a negative correlation between Wi-Fi signal strength and distance. As shown in the figure, the distance between the user's mobile phone and Wi-Fi is equal to the distance between the cars plus the length of the car, and the distance between the first car and the second car from the Wi-Fi is one car length plus the distance between the cars. Generally, the length of a family car on the market is about 4 meters to 5.5 meters. The signal strength difference caused by this distance difference is huge. Therefore, it is not possible for two users to detect the same Wi-Fi signal strength.

**Figure 22:**
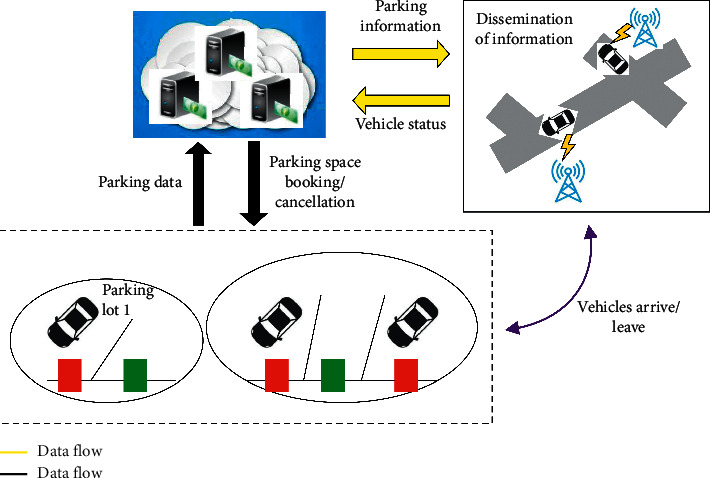
Urban parking process.

The entire urban parking process can be represented as a circular ecosystem, as shown in Figure 22, which consists of three parts and two processes. Among them, the three parts are parking users, parking lots, and parking space recommendation systems. Contains the following entities, parking drivers, vehicles, and various sensors on them, parking lot management systems and parking space detection equipment, and parking space recommendation systems. The two processes are data flow and traffic flow. The traffic flow occurs on the road where the driver is looking for a parking space and when the vehicle arrives or leaves the parking lot, which is represented by the yellow double-headed arrow in Figure 22; the data flow occurs during parking. The process of interaction between the user and the parking lot and the parking space recommendation system is represented by a black arrow in Figure 22. The parking space sensor or parking lot management system installed on the roadside uploads the collected real-time parking space data to the parking space recommendation system. The driving vehicle uploads its real-time position, speed, and other status data, and the parking space recommendation system processes the data into effective information, release information on the availability of parking spaces in each parking lot, drivers can obtain the latest parking information from smart terminals that exchange information with base stations and other transceiver platforms, and turn to the parking area they want, and then park. When the vehicle arrives or leaves the parking space, the parking space sensor updates the parking space available information and uploads the information to the driver looking for the parking space through the intelligent terminal. Considering that when there are many drivers looking for parking spaces in a certain area, they can all receive parking space availability information. It is very likely that competition for a certain free parking space resource will result in parking conflicts. Therefore, the system should have a reservation mechanism. The reservation mechanism includes the reservation of free parking spaces, the cancellation or recycling of reservation orders, etc. The reservation operation causes data flow between the driver, the parking lot, and the parking space recommendation system.

**Figure 23:**
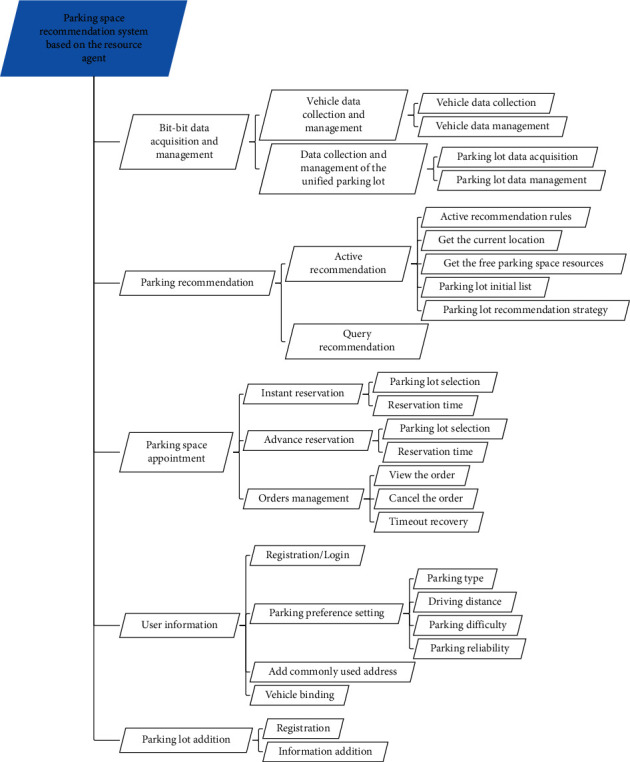
System data function structure.

Recommending suitable parking lots and parking spaces for drivers with parking needs so that they can quickly find their own satisfactory parking spaces is the original intention of this research. From the above analysis and combined with the driver user use case diagram, it can be seen that the parking space recommendation system must have parking lot recommendation, parking space reservation, and order management after reservation. However, the realization of a parking application software for driver users requires some other functions, such as system login/registration, user information addition, etc. to be more complete. In addition, the analysis of the circular ecosystem formed by the above-mentioned urban parking process shows that the realization of the parking space recommendation system requires two key steps, namely, real-time data collection and management and parking-related services. Real-time data collection and management includes the collection and management of parking space data and vehicle data. Among them, data collection is the basis for the system to provide parking-related services. However, due to the different parking detection technologies used in the current parking lot, the parking lot application has not been unified. There is no unified standard for the data interface and exchange mechanism. The heterogeneity of parking space data makes the current parking application have a strong closedness, which has great limitations in data collection and use, and it is impossible to obtain more information. Comprehensive parking information provides users with good parking recommendations. At the same time, there are many types of user vehicles, and there are many types of sensors on them. Even sensors of the same type may have different data formats and transmission methods among vehicles, making it difficult to use vehicle data. The system needs to provide a processing method for raw data so that various heterogeneous data can be represented in the same way to support parking-related services. Therefore, the system should also have real-time data collection and management functions. In summary, the functions of the parking space recommendation system based on resource agents in this subject are proposed. The system function structure is shown in Figure 23.

## 5. Simulation Experiment and Result Analysis

In order to test the application performance of the combined optimization model for multisource traffic information collection designed in this paper, software debugging and simulation experiments are carried out. The multisource traffic information collection combination optimization model provides users with a simple and unified system call interface. The packet size is set to 24 kB/s, the weighting factor *w* = 2.19, the traffic information collection time is 1025, and the size of combined sequence training group is 2100 parameters.

**Figure 24:**
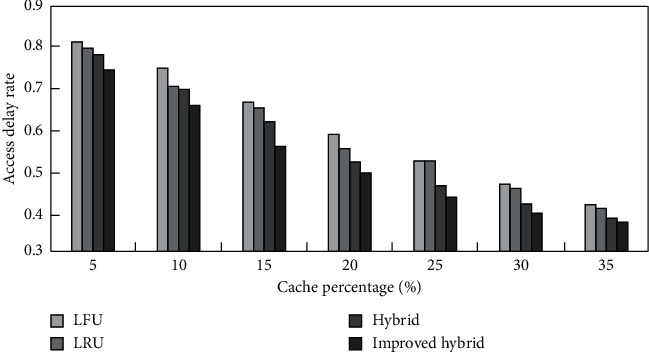
is the access delay rate of the traffic information collection combination in various cache percentages.

According to the analysis results in Table 1, multisource traffic information collection is combined, as shown in Figure 24, to test the access delay rate of traffic information collection combinations in various cache percentages.

**Figure 25:**
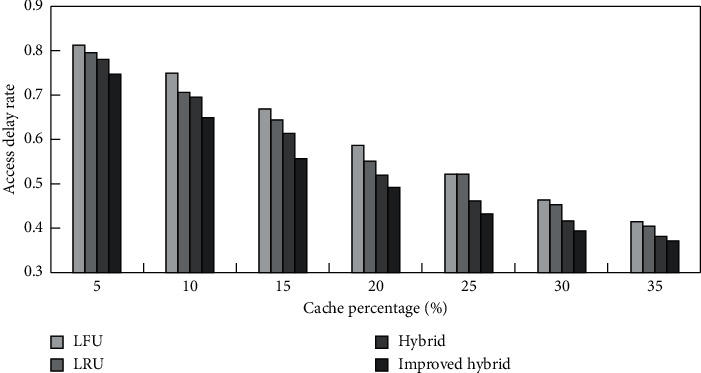
Access delay rate of traffic information collection combinations under various data sizes.

According to Figure 24, the access delay rate of the traffic information collection combination using this method is low. The delay rate of the traffic information collection combination of various data scales is tested, and the test results are obtained as shown in Figure 25.

After analyzing the above simulation results, it is found that using this method, the combined accuracy of multisource traffic information collection is high, the degree of information fusion is improved, and the delay is low.

## 6. Conclusions

In this paper, the combination ability of dynamic traffic information is improved. According to the improved information data, on the basis of collecting multisource traffic information, an information data processing system is developed combined with genetic algorithm. The multidimensional nonlinear joint statistical methods can be used to multiperform multidimensional feature space combination in the complex traffic network environment. In order to construct a multisource traffic information combination model, the genetic algorithm can be used to effectively collect the original traffic data information. According to the high-dimensional feature quantity, the obtained multisource traffic information is fused and combined to carry out the distributed clustering of traffic information and the effective control of fuzzy directivity constraints. Finally, the combined optimization model of traffic information collection has a high degree of intelligence, accurate information collection, and low delay in this paper.

## Figures and Tables

**Table 1:** System parameter settings.

Dispatching data	Number of tasks	Percentage of dynamic traffic collection combination (%)
33	1–6	55
41	4–33	44
51	33–51	33
56	51–61	21

## Data Availability

The labeled data set used to support the findings of this study are available from the corresponding author upon request.
